# Safety of Exogenous Recombinant Human Platelet‐Derived Growth Factor‐BB (rhPDGF‐BB) for Medical and Cosmetic Applications: A Review

**DOI:** 10.1111/jocd.70636

**Published:** 2025-12-30

**Authors:** Christopher K. Hee, Herbert B. Slade, Samuel E. Lynch

**Affiliations:** ^1^ Lynch Regenerative Medicine Franklin Tennessee USA; ^2^ Chisholm Clinical Research Services, LLC Ft. Worth Texas USA

## Abstract

**Background:**

Recombinant human platelet‐derived growth factor‐BB (rhPDGF‐BB or pure PDGF) has been extensively studied in medicine, resulting in four FDA approvals for products based on pure PDGF for tissue regeneration applications, including stimulation of the healing of skin wounds in diabetic patients, stimulation of healing of intra‐oral tissue defects, and stimulation of bone and tissue regeneration following foot and ankle arthrodesis surgery. More recently, pure PDGF has also been introduced for esthetic and cosmetic applications.

**Objective:**

Here, we review the extensive nonclinical and clinical pharmacokinetic and safety studies performed on rhPDGF‐BB across diverse administration routes, including topical, injection, and surgical implantation to establish a large margin of safety.

**Results:**

Pharmacokinetic studies confirmed that systemic exposure to rhPDGF‐BB is negligible following repeated topical application, injection, or implantation, minimizing any potential for systemic risks. Biocompatibility and toxicology studies demonstrated that rhPDGF‐BB applied topically to skin, implanted, or injected subcutaneously, intradermally, intramuscularly, intraperitoneally, or intravenously, one time, or repeatedly, is noncytotoxic, nonirritating, nonsensitizing, nonmutagenic, nonclastogenic, noncarcinogenic, and nontoxic. No risk of cancer incidence or cancer mortality was detected following injection, implantation, or repeated, daily topical application of rhPDGF‐BB onto partial or full thickness skin wounds for up to 20 weeks, that is, 140 doses.

**Conclusion:**

The totality of the data demonstrates that the use of rhPDGF‐BB for regeneration and rejuvenation of skin and other tissues has an excellent safety profile, a finding further affirmed by its record of safe and effective use in multiple medical indications for over 25 years.

## 1 Introduction

Platelet‐derived growth factor (PDGF) orchestrates vital processes involved in tissue regeneration, rejuvenation, and wound healing [1, 2]. The ability of PDGF to enhance these pathways has led to FDA approval of four products containing recombinant human pure PDGF BB (rhPDGF‐BB or pure PDGF) used to treat a myriad of regenerative needs [3, 4, 5, 6, 7]. Pure PDGF has also recently received rapidly increasing use in medical esthetics. Given its rapid rise in popularity, questions have been raised regarding what studies demonstrate its safety for use in esthetic medicine. As with any product, the evaluation of safety should be a prerequisite to understand the potential risk for a given use. This review summarizes the extensive record of safe and effective use of rhPDGF‐BB in medicine, highlighting those studies with the most relevance to the dermatology, plastic surgery, and medical esthetics providers, thereby providing a comprehensive source of safety information for pure PDGF to which health care providers can refer.

The four products containing rhPDGF‐BB as the active ingredient that have been approved by the FDA following rigorous pre‐clinical, pilot, and pivotal studies (medical device), and phase I–IV clinical trials (pharmaceutical/biologic) are briefly summarized in Table 1. The first rhPDGF‐BB containing product to be approved by the FDA was REGRANEX gel, a carboxymethylcellulose gel containing 0.01% (100 μg/g) rhPDGF‐BB indicated for daily treatment of chronic skin wounds for up to 20 weeks (140 daily topical applications) in the lower extremities of diabetics [4]. REGRANEX has been used safely and effectively to promote healing of these skin wounds for over 25 years as of this writing. GEM 21S (Growth Factor Enhanced Matrix) was approved by the US FDA in 2005 to promote the regeneration of intra‐oral tissues including maxillary and mandibular bone and gingiva [5]. Unlike REGRANEX, GEM21S is composed of rhPDGF‐BB (0.3 mg/mL in 20 mM sodium acetate) and a synthetic beta‐tricalcium phosphate (β‐TCP). Given the efficacy of REGRANEX and GEM21S in the field of skin wound healing and tissue regeneration, rhPDGF‐BB was also developed for orthopedic tissue regeneration applications. AUGMENT Bone Graft was approved by the FDA in 2015 as an alternative to autograft in arthrodesis (i.e., fusion procedures) of the ankle and/or hindfoot [6]. AUGMENT Bone Graft consists of rhPDGF‐BB (0.3 mg/mL in 20 mM sodium acetate) with a bioresorbable synthetic bone matrix (β‐TCP). An additional rhPDGF‐BB containing product, AUGMENT Injectable was approved by the FDA in 2018 as an alternative to autograft in arthrodesis (i.e., surgical fusion procedures) of the ankle (tibiotalar joint) and/or hindfoot [7]. AUGMENT Injectable contains rhPDGF‐BB (0.3 mg/mL in 20 mM sodium acetate) and β‐TCP as well as Type I bovine collagen matrix. More recently, pure rhPDGF‐BB has been used in the esthetic cosmetic indications. ariessence pure PDGF+ is listed with FDA under cosmetic product listing requirements and combines rhPDGF‐BB (International Nomenclature of Cosmetic Ingredients designation: SH‐polypeptide‐59) with uncrosslinked hyaluronic acid (HA) solution.

**TABLE 1:** Summary of FDA approved rhPDGF‐BB containing products.

Product	Composition	Dose	Dosage	Route of administration	FDA status
REGRANEX	0.01% (100 μg/g) rhPDGF‐BB in a carboxymethycellulose gel	6.25 μg of rhPDGF‐BB applied per cm^2^ of ulcer surface (up to 87.5 mg total dose)	Daily (for up to 20 weeks)	Topical	BLA 103691
GEM 21S (Growth Factor Enhanced Matrix)	0.3 mg/mL rhPDGF‐BB + β‐tricalcium phosphate	0.150 mg rhPDGF‐BB + 0.5 cc β‐TCP matrix	One time	Implanted	PMA P040013
AUGMENT Bone Graft	0.3 mg/mL rhPDGF‐BB + β‐tricalcium phosphate	Up to 2.7 mg rhPDGF‐BB + 9 cc β‐TCP matrix	One time	Implanted	PMA P100006
AUGMENT Injectable Bone Graft	0.3 mg/mL rhPDGF‐BB + β‐tricalcium phosphate/bovine Type I collagen matrix	Up to 0.900 mg rhPDGF‐BB + 1 g β‐TCP/bovine Type I collagen matrix	One time	Injected (14‐gauge needle)	PMA P100006 S005
Ariessence pure PDGF+	0.3 mg/mL rhPDGF‐BB + 1.2% uncrosslinked hyaluronic acid	0.150 mg rhPDGF‐BB + 18 mg HA	One time	Topical	Listed Cosmetic 53‐171423‐311193

Throughout the three decades of research on the safety and efficacy of pure PDGF for these numerous and diverse therapeutic indications, extensive studies have been conducted to evaluate the safety of pure PDGF using accepted standardized nonclinical models and randomized, prospective, blinded controlled clinical trials. This review aims to provide an assessment of the safety studies of rhPDGF‐BB that have led to its strong track record of safe use in medicine over two decades.

## 2 Pharmacokinetics of rhPDGF‐BB

PDGF is a locally acting growth factor, released at the site of injury, and interacts with the local cells through the PDGF receptors present on multiple cell types of mesenchymal embryonic origin [8, 9]. Binding of rhPDGF‐BB to the alpha and beta PDGF receptors results in receptor activation, followed by internalization of the ligand‐receptor complex into endosomes. The PDGF‐receptor complex is then dissociated, and the receptor is recycled to the cell membrane, or the PDGF‐receptor complex is degraded [8, 9]. Excess rhPDGF‐BB at the site is sequestered by endogenous mechanisms (e.g., α2‐macroglobulin), resulting in a decrease in the local concentration and limiting any potential for overstimulation of cells [10, 11]. As a result of these natural regulatory mechanisms, the actions of PDGF are limited to the local site of application.

The pharmacokinetics of exogenously added rhPDGF‐BB have been well studied. Systemic pharmacokinetic studies in rats, dogs, and baboons have demonstrated that the half‐life of PDGF is short, with an observed range of 2–55 min, even with intravenous doses of 23 times the max dose used in AUGMENT Bone Graft (2.7 mg rhPDGF‐BB), and over 400 times the dose of pure PDGF most often used in esthetic applications [12, 13]. An additional study evaluating the release of rhPDGF‐BB when applied onto the calvaria similarly showed transient retention of rhPDGF‐BB at the site of application, with a rapid loss of approximately 50% of the rhPDGF‐BB from the surgical site during the first 30 min after implantation, followed by a more gradual decrease over the next 72 h, with approximately 10% remaining at 72 h [14]. Clinical PK studies of REGRANEX showed similar findings, wherein either daily topical application onto skin or a one‐time implantation resulted in no detectable systemic absorption of the PDGF, with systemic levels similar to baseline levels in a majority of subjects [13]. Taken together, both nonclinical and clinical PK studies indicate that rhPDGF‐BB acts locally and transiently and systemic bioavailability is very low. Regardless of the route of administration, systemic exposure to PDGF is minimal and of short duration.

## 3 Safety Studies of rhPDGF‐BB

In vitro, pre‐clinical and clinical studies have been performed in order to establish the safety profile of rhPDGF‐BB across a variety of different cellular and organ systems, including skin, subcutaneous, and bone tissues.

### 3.1 Genotoxicity and Mutagenicity Assays

In vitro studies have consistently demonstrated that rhPDGF‐BB is nonmutagenic, nonclastogenic, and nongenotoxic. Specifically, bacterial mutagenicity assays (Ames test) have demonstrated exposure to rhPDGF‐BB, even at the highest concentration tested (1 mg/plate), was nonmutagenic, regardless of whether there was metabolic activation or not as part of the assay design [15, 16]. These findings extend to mammalian cell culture as well. Treatment of Chinese Hamster Ovary (CHO) cells and mouse lymphoma cells treated with rhPDGF‐BB at 2 mg/mL did not elicit a mutagenic response in these cells [16]. The genotoxic effects of rhPDGF‐BB have also been studied. Analysis of 3H‐thmidine incorporation to measure unscheduled DNA synthesis in primary rat hepatocytes exposed to rhPDGF‐BB revealed no differences compared to controls [16]. While modest decreases in cell viability were reported, it was only observed at the highest tested concentrations. An in vivo bone marrow micronucleus assay also demonstrated that rhPDGF‐BB was nongenotoxic when administered intraperitoneally for three consecutive days at doses up to 50 mg/kg [16].

### 3.2 Dermal and Ocular Irritation Assays

A variety of pre‐clinical models evaluating the effects of rhPDGF‐BB on skin revealed minimal skin irritation. Intradermal injections with rhPDGF‐BB resulted in no signs of skin irritation including erythema, edema, or eschar formation [15]. Of note, slight irritation was reported when rabbits were injected intradermally with neat, undiluted rhPDGF‐BB; however, this was expected given the chemotactic and mitogenic effects of PDGF in mesenchymal cells [15]. Additional studies applying rhPDGF‐BB topically to skin for 14 days further confirmed the absence of dermal irritation following rhPDGF‐BB application [16]. In addition to skin irritation, a rabbit ocular irritation study also showed no irritation for 110 μg/g rhPDGF‐BB gel. Finally, two clinical studies in which 30 μg/g rhPDGF‐BB gel was applied to either intact or abraded skin showed no increase in irritation relative to saline. Taken together, the pre‐clinical and clinical studies demonstrate that rhPDGF‐BB treatment does not result in irritation.

### 3.3 Sensitization Assays

The potential for rhPDGF‐BB to elicit a hypersensitivity response in pre‐clinical models was evaluated in guinea pigs exposed to rhPDGF‐BB and calcium phosphate matrices. No clinical signs of systemic toxicity, sensitization, or an allergic response were reported when rhPDGF‐BB was injected intradermally followed by a delayed topical challenge [16]. In a separate study, rhPDGF‐BB administration was found to be sensitizing when administered in the presence of Freund's complete adjuvant; however, this result is not unexpected for a recombinant human protein tested in an animal system [16]. Clinical sensitization studies in 25 subjects confirmed that rhPDGF‐BB did not induce contact sensitization following repeat topical administrations.

### 3.4 Acute, Chronic, and Repeat Dose Toxicity Studies

Pure PDGF has also been studied in both acute and chronic toxicology studies. Acute injections (up to 100 mg/kg intravenous or intraperitoneal) with rhPDGF‐BB demonstrated a significant margin of safety in mice [15, 16]. Likewise, no adverse events were detected in other pre‐clinical models including rats and monkeys at doses up to 3 mg/kg. In these studies, rhPDGF‐BB injection exhibited a low potential for acute toxicity, with the dose tested in these studies being significantly higher than clinical doses [15, 16]. Additional repeated dose studies have been performed in pre‐clinical models under a variety of routes of administration and doses. Intravenous injection of mice with rhPDGF‐BB revealed the no observed adverse effect level (NOAEL) dose was 0.3 mg/kg/day [16]. This compares to approximately 0.0025 mg/kg for one day in a typical cosmetic application. In a rabbit topical administration study, rhPDGF‐BB at 25, 76, or 256 μg/kg (357, 1077, and 3617 μg/m^2^, respectively) or placebo was applied to one intact and one abraded site on the dorsal trunk [16]. No clinical signs of systemic toxicity or deaths were observed following 3 weeks of topical application. In dose ranging studies (30, 100, or 300 μg/kg) in monkeys, mild to moderate clinical changes were reported, with the highest dose group displaying the most prominent changes. However, a recovery trend toward baseline values was apparent by day 22 of the study [16]. In other studies, monkeys were injected with a range of doses (1.5, 15, or 150 μg/kg) of rhPDGF‐BB for 13 weeks followed by a 2‐week recovery period. Even with this exaggerated dose (approximately 60 times higher than the typical topical cosmetic application) given repeatedly for 13 weeks, this study found no apparent signs of systemic toxicity such as changes in body weight, rectal temperature, blood pressure, clinical pathology parameters, or ophthalmoscopic or electrocardiographic examinations were observed. Additionally, no histopathologic abnormalities were observed between placebo and rhPDGF‐BB treated monkeys.

Longer exposure studies have been conducted in rat models. In addition to the overall toxicity measures, carcinogenicity was specifically evaluated over 30, 180, and 365 days in rats implanted with 0.3 mg/mL rhPDGF‐BB combined with β‐TCP. No toxic effects related to rhPDGF‐BB administration were observed in local microscopic findings or systemic evaluations (body weight, urinalysis, bone marrow parameters). Additionally, there were no test article–related neoplastic microscopic observations noted in either sex on day 365 and none of the animals treated with the test article were positive for anti–PDGF‐BB antibodies [13].

### 3.5 Reproductive Toxicology and Teratology Studies

The potential reproductive toxicity of rhPDGF‐BB has also been evaluated in gravid rats administered daily IV injections over 21 days of gestation using rhPDGF‐BB doses of either 40 or 400 μg/kg/day. No treatment‐related mortality or significant adverse effects were observed and none of the assessed uterine parameters were affected in all treatment groups. Fetal weights and the incidence of litters and fetuses with major malformations, minor external and visceral anomalies, was unaffected by treatment. Additionally, the plasma levels of rhPDGF‐BB in all dams and fetuses were not detectable and no anti–rhPDGF‐BB antibodies were detected in the 45 samples tested except in one pretreatment sample of one of the dams. The NOAEL for maternal toxicity and embryo fetal development reported was 400 μg/kg/day [15, 16].

### 3.6 Local Response Studies

Finally, pre‐clinical models have been utilized to evaluate the local response of rhPDGF‐BB treatment. In an intramuscular implantation study, extracts of rhPDGF‐BB combined with either β‐TCP or collagen/β‐TCP matrices were performed in rabbits to evaluate acute soft tissue responses to the test articles. Each test article elicited a mild inflammatory response involving small numbers of polymorphonuclear leukocytes, macrophages, giant cells, and fibroblasts associated with the test article implant. This tissue response was mild and similar to that of the negative control, indicating that there was not a significant biological response following intramuscular implantation. In a repeat injection study onto bone [6, 15, 16], with rhPDGF‐BB injected at the metatarsi and femora for 14 days, followed by a 6‐week washout period, temporary swelling was observed at the metatarsal injection sites, but not the femoral injection sites, with the soft tissues overlying the injection sites returning to normal appearance by 8 weeks. At Day 14, no soft tissue or bone reactions were evident in the 10 or 30 μg/mL rhPDGF‐BB dose groups. A mild inflammatory reaction at the metatarsus and a mild bone reaction consisting of osteogenesis and fibroplasia in the periosteal cortex was observed on day 14 in rats that received 100 μg/mL rhPDGF‐BB. This response was resolved by the 8‐week time point, underscoring the reversibility of the tissue response to rhPDGF‐BB when its administration is discontinued.

Based on the totality of these comprehensive nonclinical studies, the potential for rhPDGF‐BB to elicit adverse reactions appears insignificant following various routes of administration, including topical application, SC, IM, or IV injection, or with surgical implantation.

### 3.7 Clinical Safety

The clinical safety of rhPDGF‐BB has also been extensively evaluated across a variety of organ systems. For example, adverse events (AE) following REGRANEX treatment were evaluated in a total of 1006 patients with diabetic ulcers of the lower extremity. Compared to either placebo gel or good ulcer care alone, patients receiving rhPDGF‐BB gel had a similar incidence of ulcer‐related adverse events (AE) such as infection, cellulitis, and osteomyelitis [17, 18]. Additionally, the incidence of cardiovascular, respiratory, musculoskeletal, and central and peripheral nervous system disorders did not differ between treatment groups. Mortality rates were similar across all treatment groups and neutralizing antibodies against rhPDGF‐BB did not develop in any of the 452 evaluable patients treated with rhPDGF‐BB gel. Based on these studies, rhPDGF‐BB gel was determined to be safe and well tolerated for the treatment of lower extremity neuropathic diabetic ulcers. Clinical studies have also demonstrated the safety of GEM21S in patients with periodontally related defects. The most frequently reported AE was study site pain, followed by a normal sequela to the surgical procedure (typically resolved within 1 week of surgery), and then headache. Importantly, no significant differences in AE were reported across the treatment groups [19]. Studies on the safety of AUGMENT Bone Graft revealed that fewer device‐related treatment emergent adverse events, fewer serious treatment emergent adverse events, and fewer serious complications were observed in the rhPDGF‐BB/β‐TCP group compared with the autograft group [20]. Seven subjects tested positive for neutralizing activity at a single visit and all subjects returned to baseline levels at the next visits. Therefore, the presence of neutralizing antibodies was transient. None of those seven subjects had any reported allergic reactions or hypersensitivity [20]. Thus, there does not appear to be a correlation between detectable anti‐rhPDGF‐BB antibodies with neutralizing activity and clinical outcomes and AEs. Similar findings were observed in clinical studies of AUGMENT Injectable, wherein subjects treated with AUGMENT Injectable had overall similar rates of treatment emergent adverse events (TEAEs), serious TEAEs, treatment‐related TEAEs, complications, and infections compared to subjects treated with autograft, when controlling for differences in AE reporting across different clinical studies [7].

### 3.8 Cancer Risk Safety Studies

Due to its role in stimulating cell proliferation, migration, and angiogenesis, PDGF and its receptor have been identified as potential targets in cancer biology [21]. As the safety of exogenously applied rhPDGF‐BB is evaluated, it is important to evaluate the differences in temporally limited application (i.e., single administration or daily dosing for weeks to months used in wound healing and tissue regeneration) compared to the aberrant, continuous, constitutive autocrine or paracrine expression of both ligands and their receptors and signaling observed in cancer cells. As described above, rhPDGF‐BB containing products are intended to be used as a single application or as a daily administration during the period of wound healing. The pharmacokinetic evaluation of these products has demonstrated that the systemic exposure to rhPDGF‐BB is minimal, even with daily dosing [8, 9, 12, 13, 14]. Furthermore, multiple in vitro and in vivo assays demonstrate that rhPDGF‐BB is nonmutagenic, even at concentrations dramatically higher than the potential maximum clinical doses [15, 16]. As a result of the long‐term use of rhPDGF‐BB containing products (available since 1997), clinical evaluations of any relationship between the use of these products and cancer have been possible. Specific summaries of the postapproval studies on REGRANEX in regard to cancer for REGRANEX have been summarized [4, 5, 6, 7] and are applicable for other rhPDGF‐BB containing products, as they represent a worst case from a dosing perspective. That is, daily dosing for weeks to months (as opposed to a single or sporadic application) and total rhPDGF‐BB delivered in higher amounts (1.5 mg rhPDGF‐BB per tube). Initial indications (up to 3 years of follow‐up) from a matched cohort study that compared cancer incidence and cancer mortality among 1622 patients dispensed REGRANEX to 2809 otherwise similar patients who were not exposed indicated a potential risk for increased mortality when dispensed 3 or more tubes of REGRANEX (each tube of REGRANEX contains 1.5 mg of rhPDGF‐BB), based on the observation of 1 cancer death in a patient dispensed 3 tubes of REGRANEX. This led to inclusion of a boxed warning being added to the REGRANEX label in 2008 solely related to the longer‐term daily use (3 or more tubes) of this product onto large open wounds in diabetic patients. Of note, the boxed warning did not include the use of 1–2 tubes (up to 3 mg) of rhPDGF‐BB (an esthetic treatment using one 0.5 mL syringe uses 0.15 mg), nor was a box warning included in the labeling of the other products containing rhPDGF‐BB. Importantly, longer term follow‐up (up to 6 years) of the high dose REGRANEX cohort showed no increased risk of cancer incidence or mortality in REGRANEX users [22]. An additional follow‐up study evaluating cancer risk associated with the use of rhPDGF‐BB for the treatment of diabetic foot ulcers was conducted by the Veterans Administration [23]. This study compared cancer rates and overall cancer mortality between 6429 patients without prior cancer who used REGRANEX and 6429 matched comparators (12858 total patients studied) followed over 11 years (1998 through 2009). The hazard ratio for cancer mortality among those who received 3 or more tubes of REGRANEX Gel relative to those who received none was 1.04 (95% confidence interval 0.73–1.48). Therefore, this study provided no evidence of a cancer risk among rhPDGF‐BB users, and did not indicate an elevated risk of cancer mortality even among these patients with long‐term daily application of rhPDGF‐BB. A third follow‐up retrospective study using medical claims from the Veteran Affairs health care database with up to 11 years of follow‐up among patients with prior cancer observed 87 cancer deaths in the REGRANEX‐exposed cohort (*n* = 477) and 340 cancer deaths in the matched comparator cohort not exposed to REGRANEX (*n* = 1756), resulting in a hazard ratio of 0.9 (95% CI, 0.7–1.2), suggesting no causative effect on incidence or mortality from cancer in the rhPDGF‐BB treated group [4, 6, 7].

Taken together, the available data about the role that PDGF plays in cancer, as well as the comprehensive nonclinical and clinical evaluations for rhPDGF‐BB containing products as they relate to cancer, demonstrate that there is no increased risk of the incidence or mortality of cancer as a result of these treatments. The removal of the boxed warning from Regranex label even as it relates to patients that may receive up to 140 daily applications of rhPDGF‐BB onto open skin wounds should reassure both prescribers and patients of the product's safety.

## 4 Conclusion

**FIGURE 1:**
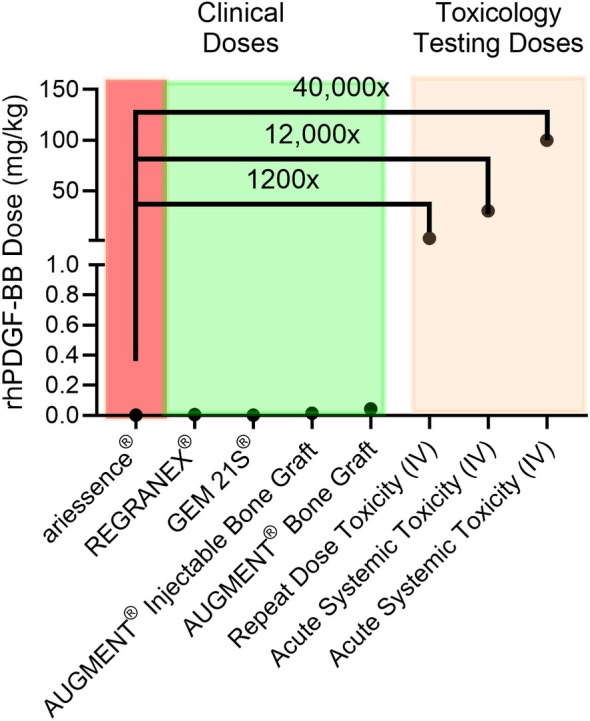
Graphical representation of doses of rhPDGF‐BB evaluated in pre‐clinical safety testing and doses used clinically.

rhPDGF‐BB has undergone extensive nonclinical and clinical safety evaluations to assess the risk associated with its use when exogenously applied topically, implanted, or injected. The totality of the results convincingly demonstrates that rhPDGF‐BB, when applied topically or injected in single or daily dosing applications, has a strong safety profile with a strong track record of safe clinical use for over 25 years. Furthermore, the amount of rhPDGF‐BB currently used in dermatology, plastic surgery and esthetic procedures is orders of magnitude below what has been demonstrated to be safe in pre‐clinical and clinical studies, further emphasizing the excellent safety profile of rhPDGF‐BB in skin (Figure 1).

## Author Contributions

C.K.H., H.B.S., and S.E.L. were responsible for conceptualization, drafting, and critical revision of the manuscript. S.E.L. supervised the drafting of the review.

## Ethics Statement

The authors have nothing to report.

## Consent

The authors have nothing to report.

## Conflicts of Interest

C.K.H. and S.E.L. are employees of and own stock and/or options in Lynch Regenerative Medicine LLC. H.B.S. is a consultant of Lynch Regenerative Medicine LLC.

## Data Availability

Data sharing not applicable to this article as no datasets were generated or analysed during the current study.
